# Structure and Mechanism of an Aspartimide-Dependent Peptide Ligase in Human Legumain**

**DOI:** 10.1002/anie.201409135

**Published:** 2015-01-28

**Authors:** Elfriede Dall, Julia C Fegg, Peter Briza, Hans Brandstetter

**Affiliations:** Department of Molecular Biology, University of Salzburg5020 Salzburg (Austria); CD Laboratory for Biosimilar ResearchUniversity of Salzburg

**Keywords:** endergonic reactions, enzyme catalysis, hydrolysis, ligases, protein modification

## Abstract

Peptide ligases expand the repertoire of genetically encoded protein architectures by synthesizing new peptide bonds, energetically driven by ATP or NTPs. Here, we report the discovery of a genuine ligase activity in human legumain (AEP) which has important roles in immunity and tumor progression that were believed to be due to its established cysteine protease activity. Defying dogma, the ligase reaction is independent of the catalytic cysteine but exploits an endogenous energy reservoir that results from the conversion of a conserved aspartate to a metastable aspartimide. Legumain’s dual protease–ligase activities are pH- and thus localization controlled, dominating at acidic and neutral pH, respectively. Their relevance includes reversible on–off switching of cystatin inhibitors and enzyme (in)activation, and may affect the generation of three-dimensional MHC epitopes. The aspartate–aspartimide (succinimide) pair represents a new paradigm of coupling endergonic reactions in ATP-scarce environments.

Primarily found in endolysosomes, legumain is involved in immunological processes such as antigen processing and TLR maturation[1] as well as in tumor progression.[2] The cysteine protease legumain can develop asparaginyl endopeptidase (AEP) and carboxypeptidase (ACP) activities in a pH- and context-dependent manner.[3] These diverse roles call for a delicate regulation of its protease activity which is in part conferred by type 2 cystatins C and F, and the most potent cystatin E/M.[4] When studying the mechanistic basis of the AEP inhibition by cystatin E/M, we found a peptide ligase activity in human AEP, apparently independent of ATP. Whereas bond-conserving modifications, such as those seen in inteins and sortases, are energetically balanced,[5] genuine peptide ligations require a source of energy.[6] Ligase and cyclase activities have been reported for plant legumains,[5b,^7^] but the mechanism of action remained unclear. These puzzling observations prompted us to crystallize human cystatin E (hCE) alone and in complex with legumain.

The crystal structure of hCE revealed the typical five-stranded antiparallel β-sheet wrapped around a central α-helix. Two disulfide bridges additionally stabilize the structure by clamping strands β4 and β5 and the hCE-specific appending structure that is inserted between strands β3 and β4 (Figure 1 a; Figure S1 and Table S1 in the Supporting Information). Parts of the appendix structure (Thr76–His82) were flexible in the electron density map.

**Figure 1:**
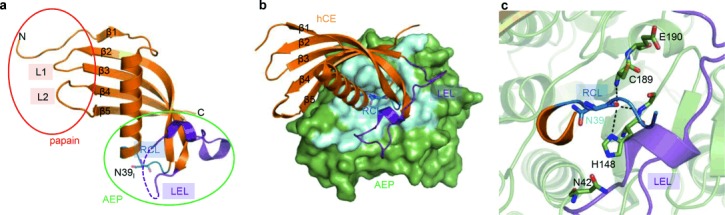
Crystal structure of human cystatin E (hCE) alone and in complex with legumain. a) Papain and AEP inhibitory sites are indicated by a red and green circle, respectively. The papain-interacting region is composed of the N-terminus and loops L1 and L2, the AEP-interacting region by the reactive center loop (RCL) harboring Asn39_I_ and the legumain exosite loop (LEL). b) The interaction of cystatin E (hCE, orange) with AEP (green) is mediated by the RCL (dark blue) and the LEL (purple). The AEP–hCE interaction surface is colored light blue. c) Enlarged view of the AEP active site reveals substrate-like binding of the cystatin RCL. Catalytic residues are shown as green sticks, Asn39_I_ as blue sticks.

The interaction with papain-like proteases has been described by the so-called elephant trunk model,[8] involving cystatin’s N-terminus (“the trunk”) and two characteristic loops L1 and L2. hCE loop L2 deviated considerably from the conformations seen in cystatins C and F (hCC and hCF) (PDB entries 3gax and 2h9, respectively; Figure 1 b). This deviation was centered around glyco-Asn108_I_ and resulted in a frameshift in the segment Pro105_I_–Met110_I_ in hCE relative to the conformation in hCC and hCF (cystatin C numbering with subscript I is used for cystatin inhibitors). We compared the affinity of glycosylated hCE and non-glycosylated (*E. coli* produced) hCE towards cathepsin B and found that the affinity of cathepsin B towards glyco-hCE (IC_50_=5.7±1.3 nm) is two times higher than towards *E. coli* hCE (IC_50_=9.3±2.4 nm).

The legumain reactive center loop (RCL) of hCE exposed Asn39_I_ in a conformation similar to that seen in hCC and hCF (Figure 1 a and Figure S1c), consistent with its suggested role for active site (P1–S1) interaction.[4a] The conserved conformation of the RCL suggests a canonical, substrate-like binding mode that is shared within the type 2 cystatin family; differences in binding affinity within hCC/E/F should be related to exosite interactions. We considered Cys73_I_–Cys83_I_ as one candidate for a legumain exosite loop (LEL) because it was stabilized relative to the reactive center loop (RCL) via charged interactions mediated by Lys75_I_ to the P2 (Ser38_I_) and P1′ (Ser40_I_) carbonyls of the RCL (Figure 1 a and Figure S1c).

In the cocrystal structure of the legumain–hCE complex we indeed found both the RCL and the LEL to contribute to AEP binding, utilizing substantial AEP contact areas that were previously found in prodomain binding within prolegumain (Figure 1 b and Figure S2a,e).[3] This observation provoked the question whether the structural mimicry between the AEP–cystatin complex and prolegumain would reflect functional analogies. One striking property of prolegumain is its stability at neutral pH, whereas isolated AEP becomes irreversibly denatured at pH>6.[9] Indeed, when complexed with either hCC or hCE, AEP remained stable at neutral pH, as shown by differential scanning fluorometry measurements (Figure S2b). The stabilization of AEP upon complexation with cystatin could also be monitored by its enzymatic activity towards a chromogenic substrate: Whereas isolated AEP became rapidly and irreversibly inactivated at pH 6.5, the preceding complex formation of AEP with hCC resulted in a basal AEP activity (Figure S2c). This cystatin-induced activity is easily explained by the continuous dissociation of AEP from the stabilizing hCC–AEP complex, thus accounting for the observed agonistic activity. A further shift from pH 6.5 to pH≤4 led to quantitative dissociation of the AEP–hCC complex, accompanied by the recovery of approximately 80 % of the initial activity (Figure S2c).

This pH profile was specific to hCC (Figure 2 a) and can be explained by the different chemical nature of the exosite loop in hCE and hCC: While the hCE legumain exosite loop (LEL) is overall hydrophobic, the hCC LEL carries characteristic charged residues, e.g., Arg70_I_ and Arg93_I_ which form salt bridges with Glu190. Consistently, charge reversal by an E190K mutation abolished the AEP–hCC, but not the AEP–hCE complex (Figure S2d).

**Figure 2:**
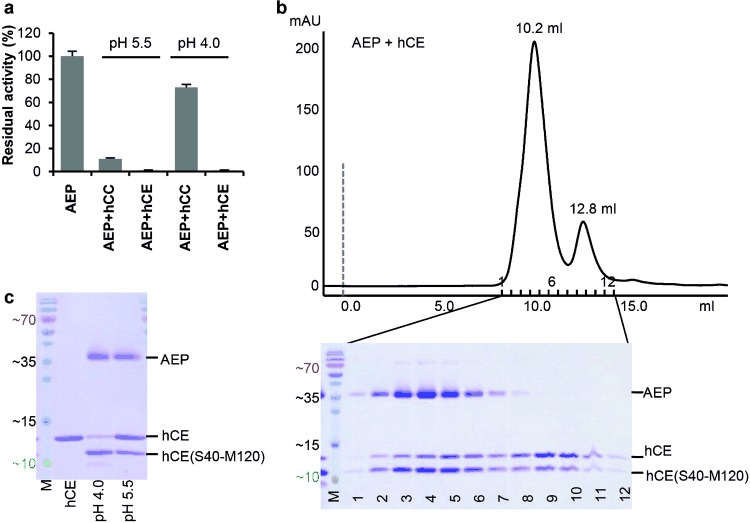
Cystatins are legumain inhibitors as well as substrates. a) Binding of cystatin C (hCC) to legumain is pH dependent and reversible at pH 4.0. Both hCE and hCC are potent AEP inhibitors at pH 5.5. Subsequent incubation at pH 4.0 had no visible effect on hCE but led to regeneration of approximately 80 % AEP activity in the case of hCC, which is indicative of inhibitor release. b) Both cleaved and intact hCE bind to legumain. Size-exclusion chromatography revealed that both intact and processed hCE co-migrate with AEP following incubation at pH 4.0. Both cystatin species were also found in a separate, monomer peak. c) Processing of hCE by AEP is pH dependent. An excess of hCE could be processed at pH 4.0 but not at pH 5.5.

We found the hCE RCL to bind to the active site in a substrate-like (“canonical”) manner, with the Asn39_I_ fully inserted into AEP’s S1 recognition site and the scissile peptide bond intact in the electron density map (Figure 1 c and Figure S2e). The geometry of the AEP active site as well as that of the hCE RCL were virtually identical in the AEP–hCE complex and in the structures of the isolated protein components, with the only exception being the catalytic Cys189 and Glu190: The thiol of Cys189 was rotated by approximately 180° and formed a zwitterionic pair with the carboxylate of Glu190, representing a resting protease state (Figure 1 c).

Canonical inhibitors often act as slowly converting substrates.[10] Therefore, we incubated AEP with a twofold molar excess of hCE and hCC at pH 4.0/5.0 for 2 h and analyzed the sample by gel filtration chromatography (Figure 2 b and Figure S3a). The corresponding elution profile was bimodal with the heterodimeric AEP–hCE complex followed by the monomeric (excess) hCE. Interestingly, partly cleaved hCE was eluted in both peaks (Figure 2 b). Mass spectrometry showed that the cystatin cleavage occurred after Asn39_I_, consistent with the proposed canonical binding mode.

Given the substrate-like binding mode of cystatin, one should expect a continuous accumulation of cleaved cystatin. Intriguingly, this was not observed. Instead, the ratio of cleaved versus intact cystatin remained largely constant over time. To better understand this puzzling behavior, we analyzed the pH dependency of hCE processing by AEP (Figure 2 c). The cleavage was favored at more acidic pH, with complete cleavage at pH≤4. This pH dependence is in conflict with the known activity profile of AEP which shows highest proteolytic activity at pH 5.5.[9] Furthermore, the processing of hCC and hCE was in marked contrast to that of family 1 cystatins (stefins A and B) where processing was progressive and occurred preferentially at Asn107_I_ and Asn61_I_ (stefin B only), and resulted in rapid degradation (Figure S3b).

In an attempt to reconcile the puzzling observations, we hypothesized that AEP could catalyze a peptide bond ligation in addition to the peptide bond hydrolysis. Thus, the incomplete cleavage of hCC/E (Figure 2 b,c and Figure S3a) would result as the pH-dependent equilibrium of two opposing reactions, with the protease activity and the ligase activity prevailing at pH 4 and pH 6, respectively.

We tested this hypothesis by preincubation of hCE/C with AEP at pH 4.0/5.0, resulting in fully cleaved hCE^†^ (or hCC^†^). When the hCE^†^ (hCC^†^)–AEP complex was incubated at different pH values, the corresponding equilibria of cleaved hCE^†^ (hCC^†^) and religated hCE (hCC) were established. Peptide bond resynthesis by AEP was most efficient at pH≥6, resulting in equimolar amounts of religated hCE (Figure 3 a and Figure S4a,b).

**Figure 3:**
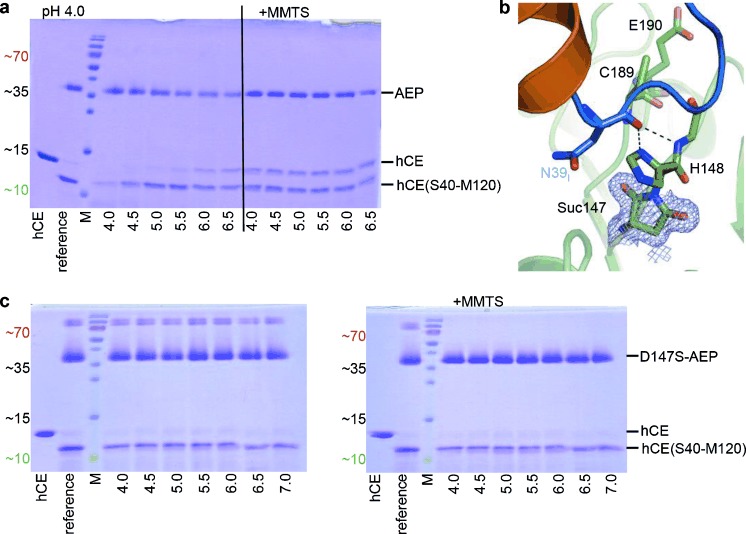
The ligase activity of legumain. a) Legumain (AEP) shows ligase activity at near-neutral pH that is not mediated by the catalytic Cys189. Upon incubation with AEP at pH 4.0, cystatin E (hCE) was completely converted to the Asn39_I_-processed form. Subsequent incubation at increasing pH values led to the reappearance of intact hCE, indicative of religation of the Asn39_I_–Ser40_I_ peptide bond. Covalent modification of the catalytic Cys189 via addition of MMTS led to pH-independent resynthesis of hCE. b) Asp147 is present as succinimide in the AEP–hCE complex. An enlarged view of the AEP (green) active site complexed with hCE (orange) is shown, with catalytic residues shown as green sticks and the hCE RCL harboring Asn39_I_ colored in blue. The electron density map (2 *F*_obs_−*F*_calc_) defining succinimide 147 (Suc147) is contoured at 1σ over the mean. c) Asp147 is essential for the religation reaction. The experiment described in (a) was repeated utilizing a combination of D147S AEP and hCE. The D147S mutant cleaved hCE, confirming its correct folding. By contrast, neither a shift in pH nor addition of MMTS led to resynthesis of hCE, demonstrating the critical role of Asp147 in the religation reaction. Analogous results were obtained with a D147G mutant.

The AEP–hCE crystal structure was determined at pH 6.5 and should thus represent the ligase state of AEP. Hereby, the thiol (SH) of the catalytic Cys189 is rotated away from the hCE peptide bond (Figure 1 c), suggesting that it is not directly involved in the ligation reaction. To investigate its role in ligation, we oxidized the Cys189 by adding *S*-methyl methanethiosulfonate (MMTS), resulting in the mixed disulfide Cys189-S-CH_3_, herein referred to as oxidized AEP, AEP_ox_. The modification was confirmed by crystallography (Figure S5a) and expectedly suppressed the AEP protease activity. Upon incubation of AEP_ox_ with cleaved hCE^†^, we observed the resynthesis to intact hCE at pH 4.0 to pH 6.5 (Figure 3 a and Figure S4a,b). This experiment confirmed that 1) the ligase and protease activities are superimposed, 2) the ligase activity of AEP is independent of Cys189, and as a consequence 3) the peptide ligase reaction is mechanistically not the reverse of protease catalysis via a thioester intermediate,[11] but must instead follow a distinct reaction mechanism. As a consequence, AEP_ox_ cannot employ an intein- or sortase-like mechanism for peptide bond ligation which would be bond and energy conserving.

Genuine peptide bond synthesis requires coupling to an energy-rich reagent that typically activates the carboxylic acid of the P1 residue.[12] In search for a suitable coupling reagent, we noted an aspartimide (succinimide) at position 147 (preceding the catalytic His148) (Figure 3 b). Succinimide can result from Asp by a condensation reaction and constitutes an energy-rich, metastable structure. Nevertheless, Asp147 was completely converted to succinimide and stabilized by the surrounding structural framework, as confirmed by crystallography and mass spectrometry (Table S2). Mass spectrometry further showed that the genetically encoded Asp147 was found in prolegumain only; upon pH-triggered autoactivation Asp147 was predominantly converted to Suc147.

Since succinimide is an established coupling reagent for amide bond synthesis,[12] we probed the role of Asp147 which is strictly conserved in all known legumains but is often a serine in caspases (Figure S5b).[13] While the legumain D147S and D147G point mutations showed (reduced) proteolytic activity and hence correct folding, they totally lacked any ligase activity (Figure 3 c). These findings demonstrate the critical role of the conjugated Asp147–Suc147 pair for the ligation reaction. Analogous to ATP, Suc147 thus pushes the endergonic peptide bond biosynthesis.[6], [14] Specifically, we propose that the O^−^ of the negatively charged carboxylate of P1 (Asn39_I_) will attack the electrophilic carbon in the proximate ketone of Suc147, resulting in a tetrahedral intermediate which undergoes ring opening (Figure 4 a,b). This P1 anhydride would energetically allow for the nucleophilic attack and aminolysis by the P1′ nitrogen, with His148 acting as a catalytic base. Sterically, however, the activated carboxylate is too distant from the P1′ nucleophile.

**Figure 4:**
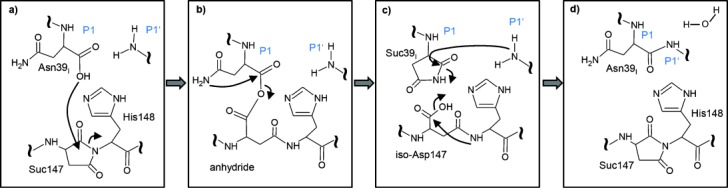
Proposed reaction scheme of the legumain ligase activity. The O^−^ of the new C-terminus of processed hCE (Asn39_I_) attacks the energy-rich Suc147, resulting in an activated carboxylic acid at Asn39_I_ (a). Next, the carbamoyl nitrogen of the P1-Asn39_I_ side chain will attack the electrophilic carbon of the carboxylic anhydride (b), thereby generating Suc39_I_ (c). The succinimide ring can now be opened via a nucleophilic substitution by the P1′ nitrogen, resulting in the intact P1–P1′ peptide bond (d). Additionally, the Suc147 may be regenerated via a condensation reaction.

Therefore, we instead propose that the conserved P1 asparagine side chain attacks the proximate electrophilic anhydride carbon, thereby releasing the single-bonded oxygen of (Iso)Asp147. This nucleophilic substitution results in another succinimide intermediate at P1 (Asn39_I_), similar to that found in inteins and asparagine lyases;[15] this P1 succinimide now allows both energetically and sterically for the attack by the P1′ nucleophile and restores the Asn side chain through amide bond formation (Figure 4 c,d). We tested this hypothesis by engineering an hCE N39_I_D point mutant. This mutant inhibited AEP protease activity and was cleaved at the Asp39_I_–Ser40_I_ bond, analogous to the wild-type hCE (Figure S5c). However, the N39_I_D mutation completely abolished the ligase activity (Figure S5d), validating its role in the peptide ligation. This conclusion is further supported by mass spectrometry data with different modifications of Asn39_I_, including deamidation (i.e. Asp39_I_), an indirect proof of a succinimide intermediate (Table S2).

Significantly, the ligase activity is not limited to cystatins as substrates. We observed peptide bond resynthesis also in cleaved prolegumain itself. Specifically, we incubated the legumain variant (D303E–D309E) at pH 5.0 to produce the characteristic autoactivation that is generated by *in trans* cleavage at Asn323–Asp324.[9] When the pH was increased to 6.0, the Asn323–Asp324 peptide bond was religated, resulting in latent prolegumain (Figure S4c). These observations are consistent with a recent report on mouse legumain.[16] The ligation rate can be estimated to be at least on the order of 1 min^−1^, as judged by an SDS-PAGE based assay (Figure S4d).

The present study reveals the mode of legumain inhibition by hCE, with the legumain RCL being distinct from the papain/cathepsin interactive site. Indeed, docking studies supported that family 2 cystatins are able to simultaneously bind to cathepsins and legumain,[4a] thereby co-localizing these important endolysosomal enzymes (Figure S6 and S7). Legumain may act as a ligase at the extracellular or cytosolic milieu, and thus silence already activated downstream proteases.[6] Equipped with a dual protease–ligase activity, legumain may also engage in *in cis* or *in trans* protein splicing, whereby a single or different target proteins are cleaved at multiple sites and recombined to a protein with new function.[17] This activity combination together with its localization to the endolysosome make legumain a critical player in the processing of antigenic peptides for MHCII presentation. While legumain’s protease activity is known to generate linear epitopes for MHCII presentation,[18] its ligase activity may link sequentially distant epitopes and thus enable the presentation of three-dimensional epitopes. Even further, legumain may silence activated MHCII complexes by religating the cleaved invariant chain Ii, thus inhibiting peptide loading. Finally, the discovery of the conjugate aspartate–succinimide pair as a proteinaceous (endogenous) energy reservoir points towards its relevance in ATP-scarce environments, e.g., in the extracellular space.
