# Supplementary material for: Structure and Mechanism of an Aspartimide-Dependent Peptide Ligase in Human Legumain
Source: Angew Chem Int Ed Engl. 2015 Jan 28;54(10):2917–21. doi: 10.1002/anie.201409135 (PMC4506564; doi:10.1002/anie.201409135)
Supplement: Supplementary file 1 [file anie0054-2917-sd1.pdf]

Angewandte Chemie
Eine Zeitschrift der Gesellschaft Deutscher Chemiker

Supporting Information
© Wiley-VCH 2015
69451 Weinheim, Germany

Structure and Mechanism of an Aspartimide-Dependent Peptide Ligase in Human Legumain**

*Elfriede Dall, Julia C. Fegg, Peter Briza, and Hans Brandstetter**

anie_201409135_sm_miscellaneous_information.pdf

SUPPORTING INFORMATION

Contents

Supplementary Figures S1 – S6	pages S2 – S10
Supplementary Tables S1 and S2	pages S11 – S12
Supplementary Methods	pages S13 – S17
Supplementary References	pages S18 – S19

Supplementary Figures

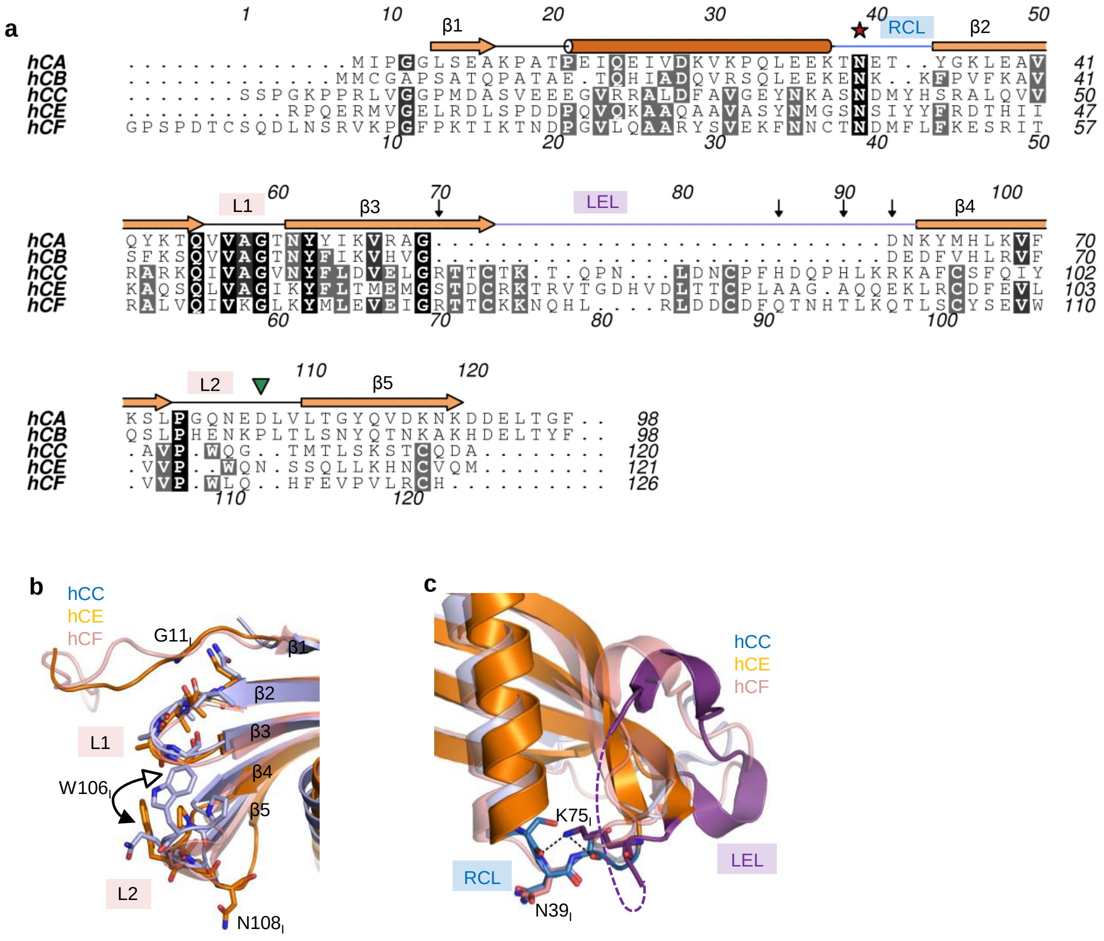

Figure S1. Comparison of cystatins.

a) Structure-based sequence alignment of cystatins A (hCA, P01040, pdb entry 3k9m), B (hCB, P04080, pdb entry 1stf), C (hCC, P01034, pdb entry 3gax), E (hCE, Q15828, pdb entry 4n6l) and F (hCF, O76096, pdb entry 2ch9). Cystatins A, B, E and F were superimposed onto cystatin C using Topmatch[1] and sequences were aligned using Aline.[2] The top sequence numbering corresponds to hCC, the bottom numbering to hCE. Black arrows mark positively charged residues on cystatin C interacting with legumain's primed side, red star: P1-Asn39$_I$, green triangle: glyco-Asn108$_I$ in cystatin E.

b) Superposition of the hCC/E/F papain interaction sites. Loop L1 of cystatin E (orange) closely resembles the conformation observed in hCC (light blue, pdb entry 3gax) and hCF (pink), loop L2 however, revealed a ~1.5 residue shift in the backbone conformation of Pro105$_I$-Ser110$_I$ as compared to Pro105$_I$ -Met110$_I$ in hCC. An arrow indicates the displacement of Trp106$_I$. The N-glycosylation site Asn108$_I$ is indicated.

c) Superposition of the hCC/E/F legumain reactive center loop (RCL). P1-Asn39$_I$ of hCE sterically resembles the conformation observed in hCC (light blue) and hCF (pink). Lys75$_I$ on the legumain exosite loop (LEL, purple) stabilizes the conformation of the RCL via hydrogen bonds to the P2 and P1' residues. A dashed purple line indicates the flexible stretch of the LEL.

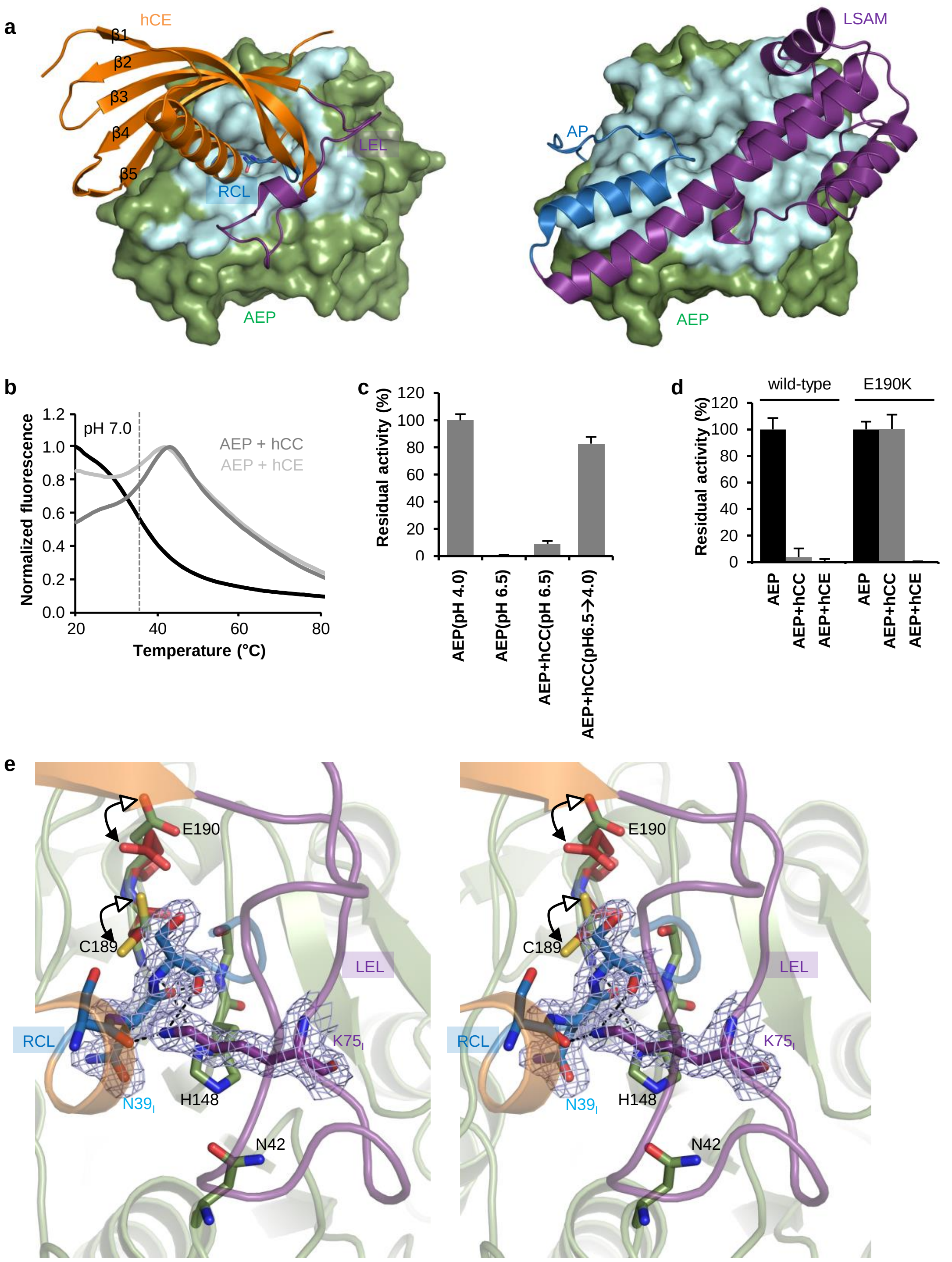

Figure S2. The legumain-cystatin interaction mimics the prodomain interaction in prolegumain.

a) Comparison of the AEP-cystatin E complex (pdb entry 4n6o) and prolegumain (pdb entry 4fgu). The AEP catalytic domain is shown in surface representation (green), surface areas that are rendered inaccessible by the cystatin or the prodomain are colored in light blue. Elements interacting with the AEP non-primed side are colored in dark blue: RCL (reactive center loop) on cystatin E (hCE) and AP (activation peptide) on prolegumain; elements interacting with the primed side are colored in purple: LEL (legumain exosite loop) on hCE and the LSAM (legumain stabilization and activity modulation) domain on prolegumain.

b) Legumain-cystatin complexes show an increased thermal stability as compared to free AEP at neutral pH. Legumain was complexed with cystatin C (hCC, dark grey) and cystatin E (light grey) at pH 5.5. Subsequently thermal denaturation curves were determined at pH 7.0 following the Thermofluor method. Free legumain served as control (black). Legumain complexed with cystatin C or E/M both led to a significant increase in T_M (indicated by a dashed line).
c) AEP activity is preserved in the cystatin complex at pH 6.5 and can be fully released at pH 4.0. Following a pH shift to 6.5 legumain was conformationally destabilized and lost its enzymatic activity [AEP (pH 6.5)] as compared to untreated legumain [AEP (pH 4.0)]. Remarkably, pre-incubation of legumain with hCC at pH 5.5, followed by a pH shift to 6.5 [AEP+hCC(pH 6.5)] resulted in a significant residual enzymatic activity, resulting from the AEP dissociation from the stabilizing complex. Upon incubation of this complex at pH 4.0, legumain activity was recovered [AEP+hCC(pH 6.5→4.0)].
d) Cystatin C does not inhibit E190K-legumain, confirming that Glu190 is a critical primed side interaction partner. Wild-type and E190K legumain (black bars) were incubated with hCC (dark grey bars) and hCE (light grey). Both hCC and hCE are inhibiting wild-type legumain. However, while hCE is also inhibiting E190K-legumain, hCC is not.
e) $Lys75_I$ is stabilizing the RCL conformation via interactions to $Ser38_I$ and $Ser40_I$. Stereo-view of the AEP (green) active site complexed with hCE. The RCL is shown in dark blue, the LEL in purple, and active site residues in green sticks. Additionally, Cys189 and Glu190 were superimposed in red sticks, as observed in a complex with a covalent peptidic inhibitor (pdb entry 4aw9). The electron density ($2F_{obs} - F_{calc}$) defining the RCL is contoured at 1σ over the mean.

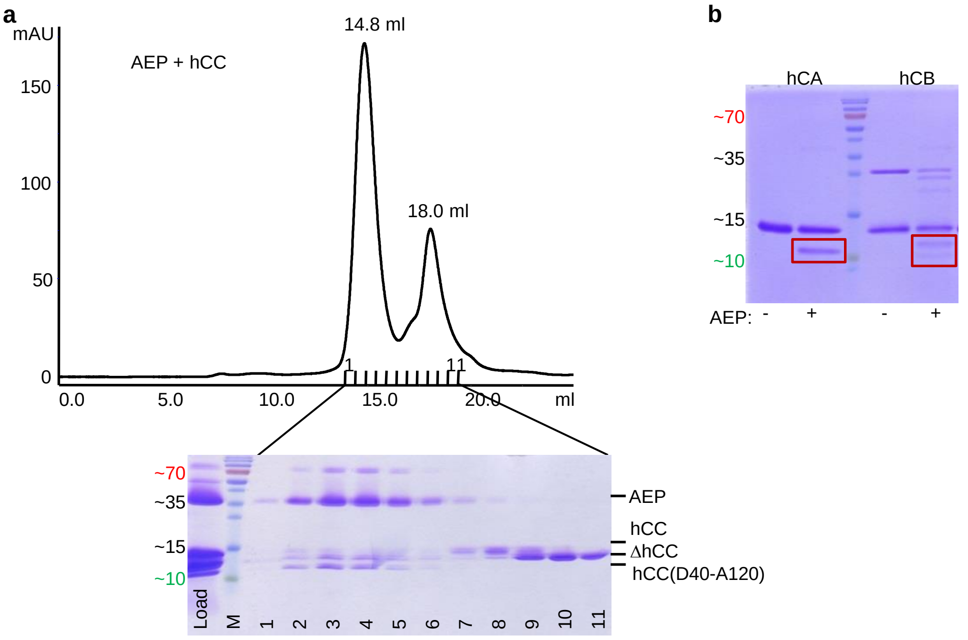

Figure S3. Cystatins as inhibitors and/or substrates to legumain.

a) Asn39$_I$ -processed cystatin C is still inhibiting legumain (AEP). When secreted from LEXSY cells, cystatin C results in two inhibitor species: full length cystatin C (hCC; S1$_I$-A120$_I$) and N-terminally truncated cystatin C (ΔhCC; L9$_I$-A120$_I$) lacking Ser1$_I$ – Arg8$_I$. The mixture of both species was incubated with legumain at pH 5.5 and subjected to size exclusion chromatography. The peak fractions 1 – 11 were analyzed by SDS-PAGE. The first peak contained legumain, both secreted cystatin C forms (hCC and ΔhCC) and an additional lower molecular weight species denoted as hCC(D40-A120) on the SDS-PAGE. Mass spectrometric analysis revealed, that hCC(D40-A120) results from cystatin C processing after Asn39$_I$. Hence, processed hCC† remains bound to legumain following cleavage at Asn39$_I$. The N-terminal truncation observed during protein expression appears not to interfere with legumain binding, consistent with the observation of both species (hCC and ΔhCC) in the complex with legumain. M: molecular weight marker, load: sample before injection.

b) Family 1 cystatins are legumain substrates, not inhibitors. Human stefin A (hCA) and B (hCB) were mixed with AEP in a 1:30 molar ratio for 10 min and progress of processing was monitored on SDS-PAGE. Mass spectrometric analysis revealed processing after Asn107$_I$ and Asn61$_I$ (stefin B only) but not Asn39$_I$. Note that stefin B partly dimerizes.

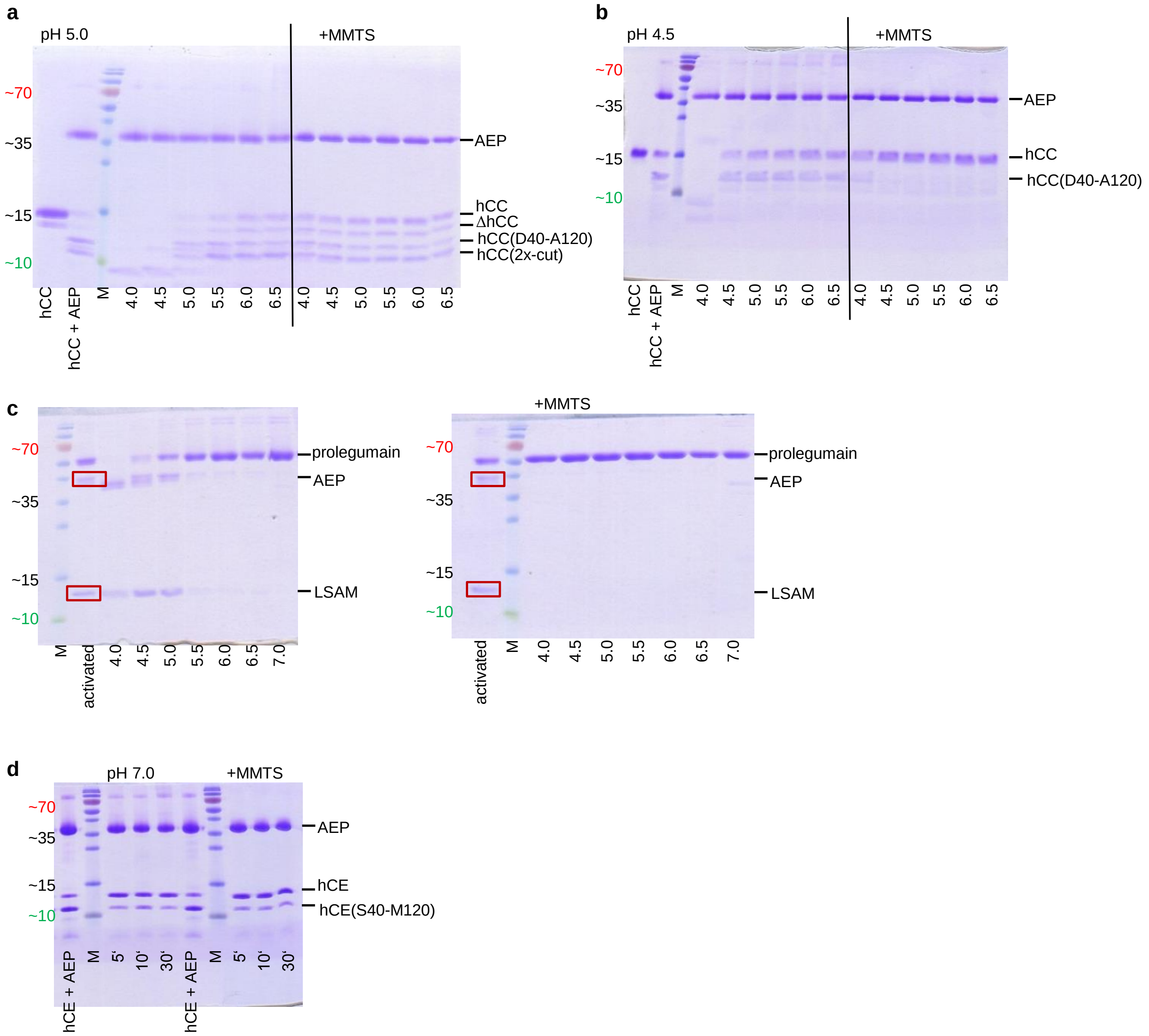

Figure S4. The ligase activity of legumain towards different substrates.
a) and b) Cystatin C (hCC) is a legumain inhibitor, protease substrate and ligase substrate. a) Cystatin C is partly N-terminally truncated when secreted from LEXSY cells and hence appears as double band (hCC = $S1_{I}$-$A120_{I}$; ΔhCC = $L9_{I}$-$A120_{I}$). A two-fold molar excess of hCC was incubated with legumain (AEP) at pH 5.0, resulting in the complete cleavage of the cystatin after the $Asn39_{I}$ residue ('hCC + AEP'). Processing becomes evident by the shifted double band 'hCC(D40-A120) and hCC(2x-cut)', corresponding to the C-terminal cleavage products of hCC. hCC(2x-cut) most likely corresponds to cystatin C that was additionally processed at a site C-terminal to the $Asn39_{I}$ processing site. The observation that legumain can cleave a two-fold excess of inhibitor implies that cleaved cystatin (hCC†) is over time released from the active site, allowing legumain to bind and cleave additional cystatin. Following incubation of the AEP-hCC† complex at increasing pH values, the secreted "unprocessed" double band reappears at near neutral pH. Additionally, by adding S-methyl methanethiosulfonate (MMTS) the catalytic Cys189 residue is covalently modified by a thiomethane group. This modification results in the stabilization of the ligation reaction (by

blocking of the hydrolysis reaction). These observations indicate (i) that both fragments hCC(S1/L9-N39) and hCC(D40-A120) remain connected also in the two chain form (hCC†) and (ii) the strict necessity of legumain for the ligation to proceed. In b) pure full-length cystatin C (S1-A120) was incubated with legumain at pH 4.5, followed by shifting pH to 4 – 6.5 with and without MMTS. In the presence of MMTS, hCC† was completely converted to unprocessed hCC.
c) Prolegumain can be resynthesized from Asn323-processed (activated) legumain via pH shift. Incubation of prolegumain at pH 5.0 results in autocatalytic processing after the Asn323 residue, resulting in the appearance of two bands corresponding to the AEP catalytic domain and the LSAM (legumain stabilization and activity modulation) domain. When the pH 5.0 activated legumain was incubated at near neutral pH, the prolegumain band accumulated and the AEP and LSAM bands correspondingly disappeared, indicative for resynthesis of the intact prolegumain. Likewise, addition of MMTS led to the stabilization of the ligation reaction. To minimize non-specific additional cleavages, a D303E-D309E mutant was used.
d) (Re)ligation of cystatin E by legumain proceeds in less than 5 minutes. In an attempt to estimate the speed of the legumain ligase activity, cystatin E was incubated with legumain at pH 4.0 (AEP + hCE) followed by a shift to pH 7.0 or the addition of MMTS (at pH 4.0). In either case processed hCE† was converted to unprocessed hCE in less than 5 minutes.
M: molecular weight marker.

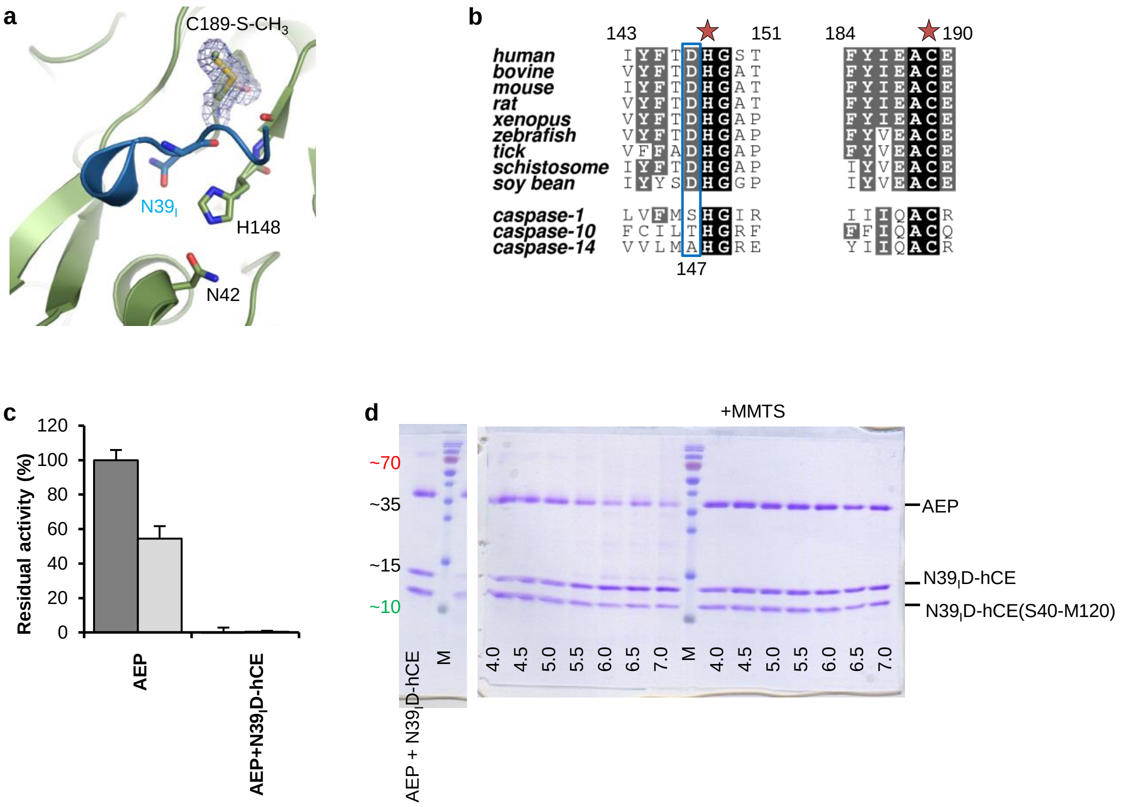

Figure S5. The ligase activity of legumain is dependent on succinimide147 and Asn39$_I$.

a) Site-specific oxidation of Cys189 by MMTS treatment. Zoom-in view on the active site of oxidized AEP (green) in complex with hCE (pdb entry 4n6n). The crystal structure of MMTS treated AEP in complex with hCE confirmed that MMTS treatment leads to the covalent modification of the catalytic Cys189 residue with a thiomethane group (C189-S-CH$_3$). The electron density (2F$_{obs}$ – F$_{calc}$) defining the C189-S-CH$_3$ is contoured at 1σ over the mean. Catalytic residues are shown as green sticks, the hCE RCL (reactive center loop) in blue cartoon. For clarity reasons, the remaining parts of hCE have been omitted from this figure.

b) Sequence alignment of legumains and caspases. Sequence alignment of human (Q99538), mouse (O89017), bovine (Q95M12), rat (Q5PPG2), schistosome (P42665), xenopus (Q6PGT1), zebrafish (Q6NYJ7), ixodes ricinus (tick, Q6PRC7) and soybean (P49045) legumain and human caspase-1 (P29466), caspase-10 (Q92851) and caspase-14 (P31944). Alignments were created using ClustalW and Aline.[2] The sequence numbering corresponds to human legumain. Red stars indicate protease catalytic residues, the ligase catalytic Suc-Asp147 in legumain is boxed.

c) N39$_I$D-hCE is an AEP inhibitor at pH 5.5 and 4.0. AEP was incubated with N39$_I$D-hCE in a 1:10 molar ratio and activity was assayed at pH 5.5 (dark grey) and 4.0 (light grey) in legumain assay buffer containing Bz-Asn-pNA substrate.

d) N39$_I$D-hCE is an AEP protease substrate but not a ligase substrate. The experiment in Figure 3a, was repeated, utilizing an N39$_I$D-hCE variant. The complex of AEP with processed N39$_I$D-hCE† was formed at pH 4.0. Neither pH shift nor addition of MMTS resulted in resynthesis of the intact inhibitor, demonstrating the critical role of Asn39$_I$ for the religation reaction.

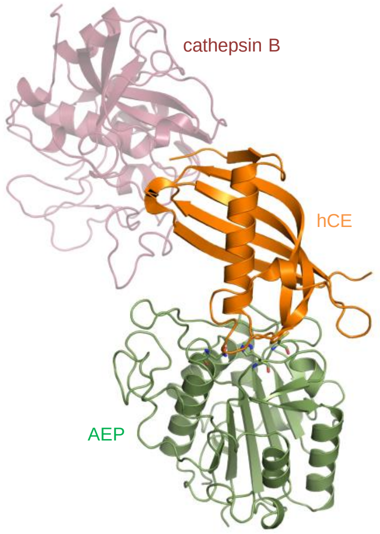

Figure S6. *In silico* docking studies confirm the simultaneous binding of cathepsins and legumain (AEP) to cystatins.

Model of a ternary cathepsin B-cystatin E-legumain complex. The model is based on a crystal structure of cathepsin B in complex with stefin A (pdb entry 3k9m). Cystatin E (hCE) of the hCE-AEP complex structure (pdb entry 4n6n) was superimposed onto stefin A. No clashes between cathepsin B and legumain were observed.

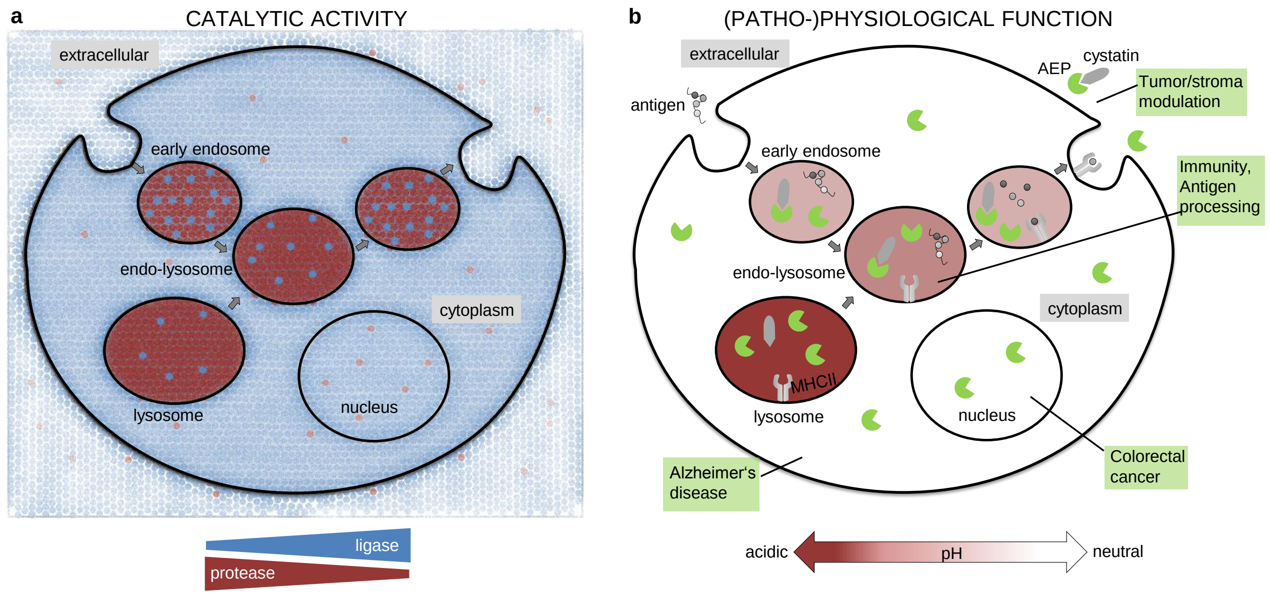

Figure S7. Legumain function is linked to its localization.
a) Legumain has been shown in different (extra-)cellular compartments with strongly varying pH, including the endo/lysosome, the nucleus,[3] the cytoplasm,[4] and the extracellular space.[5] Whereas the classic cysteine protease activity prevails in the endolysosomes (red), the ligase activity is likely to dominate in most other environments (blue), albeit substrate dependent.
b) Trafficking of legumain is accompanied by specific molecular complexes such as $\alpha_V\beta_3$ integrin and type-2 cystatins. Binding of cystatin C to legumain is pH dependent and has a stabilizing effect, suggesting a molecular recycling mechanism. At pH > 4.5 legumain binds cystatin C (hCC) and cystatin E (hCE) alike. A binary or ternary complex with cathepsins may form intra- or extracellularly. Legumain is stabilized in the complex with hCC at the extracellular pH environment. Internalization of a legumain-hCC complex, possibly by another cell, releases free and active legumain in acidic compartments; legumain could thus again function in antigen processing. Cystatin E on the other hand may serve as an intra- and extracellular inhibitor of legumain.

Table S1. X-ray data collection and refinement statistics.

	hCE	glyco-hCE	AEP-hCE ($C189-S-CH_3$)	AEP-hCE
**Data collection**				
Space group	$P2_12_12$	$P12_11$	$P12_11$	$P12_11$
Cell dimensions				
a, *b*, *c* (Å)	42.7, 68.6, 40.28	39.7, 82.6, 43.4	44.6, 85.7, 58.9	44.5, 85.5, 58.9
α, β, γ (°)	90, 90, 90	90, 112, 90	90, 94, 90	90, 94, 90
Resolution (Å)[a]	68.6–1.9(2.0–1.9)	41.3–2.3(2.4–2.3)	48.4–1.8(1.9–1.8)	48.3–1.8(1.9–1.8)
R_{merge}	0.15(0.53)	0.10(0.35)	0.06(0.25)	0.10(0.75)
$I/\sigma I$	6.5(3.0)	7.2(2.6)	9.5(3.4)	9.9(3.0)
Completeness (%)	98.9(98.8)	95.5(96.4)	97.7(90.7)	99.9(100)
Redundancy	4.7(5.0)	3.5(3.0)	2.5(2.3)	3.6(3.7)
**Refinement**				
Resolution (Å)	36.2–1.95	40.1–2.9	44.5–1.87	44.4–1.80
No. reflections	8927	5266	34006	38725
R_{work} / R_{free}	21.1/23.9	26.0/28.3	21.3/21.9	20.8/23.0
No. atoms				
Protein	898	1827	3026	3008
Ligand/ion	0	5	72	72
Water	91	29	244	279
Overall B-factors (Å^2)	25.5	33.8	21.5	24.4
R.m.s deviations				
Bond lengths (Å)	0.005	0.004	0.004	0.004
Bond angles (°)	0.94	0.81	0.98	0.94

Each structure was determined from a single crystal.

[a] Highest resolution shell is shown in parentheses.

Table S2. Mass spectrometric analysis confirming Asp/Suc147 and Asn39$_I$ deamidation.

Sample	Peptide sequence	Calculated mass	Measured mass
**AEP + hCE**			
AEP S135 – N158	SGPQDHVFIYFTDHGSTGILVFPN	2647.27	2647.2708
	SGPQDHVFIYFTDHGSTGILVFPN (-H_2O)	2629.26 (-18.01)	2629.2600
hCE M36$_I$ – R45$_I$	MGSNSIYYFR	1236.56	1236.5597
	MGSNSIYYFR (N→D)	1237.54 (+0.98)	1237.5437
**AEP + YVAD-cmk**			
AEP S135 – K164	SGPQDHVFIYFTDHGSTGILVFPNEDLHVK	3368.65	3368.6465
	SGPQDHVFIYFTDHGSTGILVFPNEDLHVK (-H_2O)	3350.64 (-18.01)	3350.6360

Supplementary Methods

Cloning

Human wild-type, E190K-, D147S/G- and N263Q-prolegumain constructs were cloned as described earlier.[6] Human cystatin C (hCC) and E (hCE) full length cDNA clones IRATp970B0214D and IRAUp969C0894D were purchased from Source BioScience (Nottingham, United Kingdom). Cystatin C lacking the N-terminal signal peptide was PCR amplified using a forward primer carrying an XbaI restriction site (ACGGTCTAGAGTCCAGTCCCGGCAAGCCG) and a reverse primer carrying a KpnI restriction site (ACGTGGTACCGGCGTCCTGACAGGTGGATTTC). Following XbaI and KpnI digestion the insert was ligated into the pLEXSY-sat2 vector (Jena Bioscience, Germany). Similarly, cystatin E was PCR amplified using primers carrying XbaI (ACGGTCTAGAGCGGCCGCAGGAGCGCATGG) and KpnI (ACGTGGTACCCATCTGCACACAGTTGTGC) restriction sites and subsequently ligated into the pLEXSY-sat2 vector. The final constructs carried an N-terminal signal peptide for secretory expression into the LEXSY supernatant and a C-terminal His_6-tag for purification.

Additionally, to produce unglycosylated cystatin E, the insert lacking the N-terminal signal peptide was PCR amplified using a forward primer harboring an NcoI restriction site (ACGTCCATGGATCGGCCGCAGGAGCGCATG) and a reverse primer harbouring an XhoI restriction site (ACGTCTCGAGCATCTGCACACAGTTGTGC). The resulting PCR product was ligated into the pET-22b(+) vector (Novagen) utilizing the NcoI and XhoI restriction sites. The expression construct carried an N-terminal signal peptide for periplasmic expression in *E.coli* and a C-terminal His_6-tag for purification. The point mutation N39D was introduced as described earlier.[6] Correctness of expression constructs was confirmed via DNA-sequencing by Eurofins MWG Operon (Martinsried, Germany).

Expression and Purification

Human prolegumain, hCC and hCE were produced using the *Leishmania tarentolae* expression system (LEXSY; Jena Bioscience, Germany) following protocols described previously.[6-7] Briefly, the LEXSY P10 host was used for stable transfection of the expression-constructs followed by selection of positive clones using nourseothricin (Jena Bioscience).[6, 8] Protein expression was carried out in shaking culture (140 rpm, 26 °C) until an OD_{600} ~3 was reached. Cells were pelleted via centrifugation and His_6-tagged protein was harvested from the supernatant by batch incubation with Ni-NTA Superflow resin (Qiagen, Hilden, Germany). Following Ni^{2+}-purification, elutions were concentrated using Amicon Ultra centrifugal filter units (MWCO: 10 kDa in case of legumain, 3 kDa in case of cystatins; Millipore) and buffer exchanged via PD-10 columns (GE-Healthcare) to get the protein in the final buffer 20 mM Tris pH 7.5, 20 mM NaCl and 5 mM DTT in case of legumain and 50 mM citric acid pH 5.5 and 50 mM NaCl in case of cystatins. Subsequently, proteins were again concentrated and subjected to size exclusion chromatography (SEC) utilizing the Äkta FPLC system equipped with a Superdex 75 10/300 GL column (GE Healthcare) utilizing the buffers also used on the PD-10 columns. For subsequent inhibition assays prolegumain was activated to the asparaginyl-specific endopeptidase (AEP) at pH 4.0 as described previously.[6]

Additionally, hCE was expressed in *E.coli* Bl21(DE3) cells. Briefly, the expression plasmid was transformed into Bl21(DE3) cells. For large scale expression, cells were grown in 2 l flasks filled with 600 ml LB medium (Carl Roth, Karlsruhe, Germany) supplemented with 100 µg/ml ampicillin at 37 °C with agitation at 220 rev/min until an OD_{600} of 0.8 – 1.0 was reached. Expression was induced at 25 °C by the addition of 1 mM IPTG (isopropyl β-d-1-thiogalactopyranoside). After overnight expression cultures were harvested by centrifugation (10 min, 4000 rpm, 4 °C). Cell pellets were resuspended in 20 ml ice-cold lysis buffer

composed of 20 mM Tris pH 7.5 and 300 mM NaCl followed by cell lysis via sonication (4 cycles with 30 s pulses at 40 W with 4 min breaks). The lysate was centrifuged twice at 17500 g for 15 min at 4 °C. The cleared supernatant containing soluble protein was batch-incubated with Ni-NTA Superflow resin for 20 min at 4 °C. Following a washing step with lysis buffer containing 10 mM imidazole, bound protein was eluted with lysis buffer containing 250 mM imidazole. As described for protein expressed in LEXSY, elutions were concentrated and buffer exchanged to 50 mM citric acid pH 5.5 and 50 mM NaCl and subsequently subjected to SEC utilizing a SUPERDEX 75 10/300 GL column (GE Healthcare) equilibrated in the buffer mentioned before.

Inhibition assays

Inhibition of wild-type legumain and E190K-legumain was tested in legumain assay buffer (50 mM citric acid pH 5.5, 100 mM NaCl) containing 0.4 mM Benzoyl-L-Asparaginyl-para-NHPhNO2 (Bz-Asn-pNA, Bachem). Assays were carried out in an Infinite M200 Plate Reader (Tecan). Briefly, the assay buffer was preincubated with 10 µM cystatin followed by the addition of 5 µM wild-type or E190K-legumain. Increase in absorbance was measured at 405 nm and 37 °C. All experiments were carried out at least in triplicate.

pH-dependent proteolysis of cystatins

To assay the pH-dependence of hCC and hCE processing by legumain, active AEP (0.2 mg/ml) was incubated with a twofold molar excess of cystatin at 37 °C in a buffer composed of 50 mM citric acid pH 4.0/5.5 and 100 mM NaCl. Progress of inhibitor hydrolysis was monitored on SDS-PAGE. Additionally, selected samples were analyzed by SEC utilizing a SUPERDEX 75 10/300 GL column in case of hCE and a SUPERDEX 200 10/300 GL column in case of hCC. The columns were equilibrated in a buffer composed of 50 mM citric acid pH 4.5/5.5 and 100 mM NaCl.

pH-dependence of complex formation

Legumain activated at pH 4.0 was incubated with hCC and hCE in a 1:2 molar ratio at pH 5.5 and 37 °C in a buffer composed of 50 mM citric acid pH 5.5 and 100 mM NaCl. In a control experiment buffer (50 mM citric acid pH 5.5, 50 mM NaCl) was added instead of cystatins. After 10 min incubation, pH was shifted to 5.5 or 6.5 utilizing a NAP-5 column pre-equilibrated with 50 mM citric acid pH 5.5/MES pH 6.5 and 100 mM NaCl. Following 10 min incubation at 37 °C pH was shifted back to 5.5 utilizing a NAP-10 column equilibrated in 50 mM citric acid pH 5.5 and 100 mM NaCl. Subsequently, samples were loaded on a SUPERDEX 75 10/300 GL column to separate complexed cystatins from free cystatin. Peak fractions were analyzed by SDS-PAGE and fractions containing stoichiometric legumain-cystatin complexes were used for activity assays. Turnover of Bz-Asn-pNA was measured in legumain assay buffer (50 mM citric acid pH 5.5, 100 mM NaCl) at 37 °C. pH-dependent dissociation of the legumain-cystatin complex was assayed via a 10 min pre-incubation of 50 µl peak fraction (containing the enzyme-inhibitor complex) at pH 4.0 via the addition of 100 mM citric acid pH 4.0.

Briefly, 45 µl assay buffer containing 0.4 mM Bz-Asn-pNA were mixed with 5 µl peak fraction and increase in absorbance was measured at 405 nm. All measurements were performed at least in triplicate.

Mass spectrometry

Legumain activated at pH 4.0 was incubated with cystatins in a 1:5 molar ratio in a buffer composed of 50 mM citric acid pH 5.5 and 100 mM NaCl at 22 °C overnight. Control experiments contained either protease only or inhibitor only in the same assay buffer.

Subsequently, samples were analyzed by SDS-PAGE and mass spectrometry, utilizing an ESI-Orbitrap setup.
Likewise, processing of stefin A and stefin B was assayed. Recombinant human stefin A and B were purchased from Sino Biological (Beijing, China). Briefly, 0.1 mg/ml of the respective stefin was incubated with pH 4.0 activated legumain in a ~1:30 (AEP : stefin) molar ratio for 10 min at 37 °C in a buffer composed of 50 mM citric acid pH 5.5 and 100 mM NaCl.
Additionally, prolegumain, pH 4.0 activated legumain, legumain in complex with hCE and hCE alone were subjected to tryptic digestion at pH ~5.0. The resulting peptides were then further analysed via mass spectrometry for the presence of aspartic acid or succinimide at position 147 on legumain or modification of $Asn39_I$ on hCE.

Ligation assays

Active legumain (0.2 mg/ml) was incubated with hCE in a twofold molar excess (0.2 mg/ml) at pH 4.0 in a buffer composed of 100 mM citric acid pH 4.0 and 100 mM NaCl. Following ~3 h incubation at 37 °C cystatin E was completely processed as confirmed by SDS-PAGE. The complex was loaded on a NAP-5 column (GE Healthcare) to get the protein in a buffer composed of 50 mM citric acid pH 5.5 and 100 mM NaCl. Subsequently, aliquots of 100 µl were subjected with 100 mM of a pH stock solution (pH 4.0 – 6.0: citric acid, pH 6.5: MES, pH 7.0: Tris) assaying a pH range from 4.0 to 7.0 in steps of 0.5 pH units. Additionally, 100 µM S-methyl methanethiosulfonate (MMTS, Sigma-Aldrich) was added. MMTS reacts with sulfhydryl-groups leading to the covalent addition of a thiomethane group. In the control experiments MMTS was replaced by DMSO. Samples were incubated at 37 °C and the progress of religation was monitored on SDS-PAGE for 2 h. The same experiment was repeated for D147S/G-AEP in combination with hCE and wild-type AEP in combination with $N39_I$D-hCE or hCC. In case of D147S-AEP, complete processing of hCE was achieved after 6 h incubation. The initial processing of hCC by wild-type AEP was performed at pH 5.0 or pH 4.5.
To test the efficiency of cystatin E religation by legumain, cystatin E was incubated with legumain at pH 4.0 until it was mostly converted to the processed form, as described above. Subsequently, pH was shifted to 7.0 by the addition of 100 mM of a pH stock solution (1 M Tris pH 7.0). Additionally, 500 µM MMTS was added in a second experiment. Both samples were incubated at 37 °C and the progress of religation was monitored on SDS-PAGE.
To test the auto-ligation activity of AEP, D303E/D309E-prolegumain was incubated at pH 5.0 (37 °C, 4 h) resulting in autocatalytic processing after the Asn323 residue, as evidenced by SDS-PAGE. Subsequently, activated legumain was incubated at different pH values with and without MMTS, following the procedure described above.

Differential scanning fluorimetry

Thermal denaturation curves of legumain-cystatin complexes were collected via the thermofluor method as described by Ericsson et al.[9] Samples investigated were pH 4.0 activated legumain and legumain in complex with hCC or hCE respectively. Legumain activated at pH 4.0 was incubated with cystatin C or E in a 1:2 molar ratio at pH 5.5 and 37 °C. After 10 min incubation, the pH was shifted to 6.5 utilizing a NAP-5 column pre-equilibrated in 50 mM MES pH 6.5 and 100 mM NaCl. Following 10 min incubation at 37 °C, the pH was shifted back to 5.5 via a NAP-10 column (50 mM citric acid pH 5.5, 100 mM NaCl). Subsequently, samples were loaded on a SUPERDEX 200 10/300 GL column (GE Healthcare) to separate complexed cystatin from free cystatin. Selected fractions were analysed by SDS-PAGE and a peak fraction containing a stoichiometric legumain-cystatin complex was concentrated to 1-2 mg/ml. Untreated AEP served as control. SYPRO Orange (Invitrogen) was added at 50x concentration and 2.5 µl of the respective solution were added to 22.5 µl buffer composed of 60 mM HEPES pH 7.0. Melting curves were collected in

a 7500 Real Time PCR System (Applied Biosystems) from 20 to 95 °C. Analysis of fluorescence data was performed as described previously.[10]

Cathepsin B assays

Recombinant rat cathepsin B was a generous gift of Lukas Mach (BOKU, Vienna). For a detailed description on cathepsin B production see Jia Z. *et al.* (2005).[11] IC_{50} values of glycosylated hCE produced in LEXSY and unglycosylated hCE produced in *E.coli* towards cathepsin B and legumain were determined using nonlinear regression routines. Basically, hCE at 0 – 900 nM concentration was preincubated in cathepsin B assay buffer (50 mM MES pH 5.0, 0.01 % CHAPS, 1 mM DTT) containing 10 µM Z-Phe-Arg-AMC (Z-FR-AMC; kindly provided by Lukas Mach) at 37 °C. The reaction was started by the addition of 10 nM cathepsin B. Inhibition of legumain was assayed at 4 nM enzyme concentration in legumain assay buffer at pH 5.5 (50 mM citric acid pH 5.5, 100 mM NaCl and 1 mM DTT). Substrate turnover was measured by using an Infinite M200 Plate Reader (Tecan). Fluorescence increase was detected at 460 nm upon excitation at 380 nm. All experiments were repeated at least three times.

Crystallization and X-ray data collection of cystatin E

For crystallization hCE produced in *E.coli* was concentrated to ~20 mg/ml. 0.2 µl of concentrated hCE were mixed with 0.2 µl screen solution (Hampton Index HT or JBScreen Classic) and equilibrated with 60 µl reservoir solution in 96-well Intelli Plates (Art Robbins Instruments) at 20 °C in a sitting drop setup. After 2 days crystals were observed in a condition composed of 30 % PEG 4000, 100 mM sodium acetate pH 4.6 and 200 mM ammonium sulfate. A native dataset was collected at 100 K on beamline ID29 (ESRF, Grenoble) equipped with a Pilatus 6M detector to a resolution of 1.9 Å. 720 images were collected at a wavelength of 0.9762 Å at 0.25 ° oscillation range and 0.08 s exposure time.
Likewise, glyco-hCE produced in LEXSY was concentrated to ~25 mg/ml and initial crystallization screening was performed at 4 °C using the JBScreen Classic. Crystals grew in a condition composed of 22 % PEG 8000, 100 mM MES pH 6.5 and 200 mM ammonium sulfate upon mixing 0.2 µl protein solution with 0.2 µl screen solution. X-ray data were collected at beamline ID14-4 (ESRF, Grenoble) at 100 K equipped with a Q315r ADSC CCD detector. Data were collected to a resolution of 2.3 Å at 1.0 ° oscillation range and a wavelength of 0.9393 Å.

Crystallization and X-ray data collection of the AEP-hCE complexes

To crystallize legumain in complex with hCE the N263Q-prolegumain variant, missing one N-glycosylation site, was activated at pH 4.0 and purified as described previously.[6, 8] Following concentration to ~1 mg/ml the protein sample buffer was exchanged to 50 mM citric acid pH 5.5 and 50 mM NaCl using a PD-10 column (GE Healthcare) to remove the reducing agent (DTT). Subsequently, legumain activity was blocked by the addition of MMTS. MMTS was added to the active enzyme at a concentration of 500 µM and the inhibition efficiency was estimated in a Bz-Asn-pNA assay. *E.coli* produced hCE was added in a 1 : 1.1 molar ratio and allowed for complex formation for ~45 min at 22 °C. Subsequently the complex was concentrated to ~30 mg/ml utilizing Vivaspin concentrators (MWCO: 10 kDa, Sartorius Stedim Biotech). Initial crystallization screening was performed in a sitting-drop vapour diffusion setup. 0.2 µl concentrated enzyme-inhibitor complex were mixed with 0.2 µl ProPlex (Molecular Dimensions) screen solution and equilibrated against 60 µl reservoir solution in 96 well INTELLI-PLATEs at 4 °C. After 3 – 5 days crystals appeared in a condition composed of 25 % PEG 4000, 100 mM MES pH 6.5 and 200 mM potassium iodide. For cryo-protection, a cryo-solution composed of the reservoir solution supplemented with 30 % glycerol was added stepwise to the drops containing crystals before flash freezing

in liquid nitrogen. To remove the thiomethane modification from the catalytic Cys189 of legumain, DTT was added to the crystals at a concentration of ~40 mM. After 72 h incubation the soaked crystals were similarly harvested in liquid nitrogen. Native x-ray data sets were collected at beamline ID14-4 at a wavelength of 0.9393 Å and 0.55 ° or 1 ° oscillation range to a resolution of 1.8 Å.

Structure solution of free cystatin E and in complex with legumain

Data processing was performed utilizing iMOSFLM[12] and SCALA from the CCP4 program suite.[13] A sequence alignment of hCC and hCE was created using ClustalW.[14] Based on this alignment, hCC coordinates (pdb entry 3gax) were mutated to the corresponding hCE residues using CHAINSAW.[15] The resulting model was then used for molecular replacement using PHASER.[16] Repeated cycles of manual rebuilding in COOT[17] and refinement using phenix.refine[18] were carried out. The resulting model was used for molecular replacement in glyco-hCE data utilizing PHASER.[16]

Datasets of AEP complexed with hCE were similarly processed. An initial model was obtained by molecular replacement using AutoMR[16] from the Phenix (Python-based Hierarchical Environment for Integrated Xtallography) program suite[19] utilizing coordinates of isolated AEP (pdb entry 4aw9) and hCE as search models. Iterative cycles of rebuilding in COOT[17] followed by refinement in phenix.refine[18] and REFMAC[20] were carried out. The final structures were analysed using PROCHECK,[21] MolProbity[22] and CDE.[23] Coordinates and structure factors were deposited with the PDB under entry codes 4n6l, 4n6m, 4n6n, and 4n6o. Pymol[24] was used to create figures illustrating structures. Complex assemblies of legumain with hCE and the AEP domain with the prodomain were analysed using the 'Protein interfaces, surfaces and assemblies' service PISA[25] at the European Bioinformatics Institute (http://www.ebi.ac.uk/pdbe/prot_int/pistart.html).

The atomic coordinates and experimental structure factors have been deposited with the Protein Data Bank (www.pdb.org) under the entry codes 4n6l, 4n6m, 4n6n, and 4n6o.

Molecular modelling

A model of hCE in complex with legumain and cathepsin B was created using Topmatch.[26] Specifically, the crystal structure of a cathepsin B-stefin A complex (pdb entry 3k9m) served as a template to align hCE in complex with legumain (pdb entry 4n6n).

Supplementary References

[1] M. J. Sippl, M. Wiederstein, *Bioinformatics* **2008**, *24*, 426-427.
[2] C. S. Bond, A. W. Schuttelkopf, *Acta Crystallogr D Biol Crystallogr* **2009**, *65*, 510-512.
[3] M. H. Haugen, H. T. Johansen, S. J. Pettersen, R. Solberg, K. Brix, K. Flatmark, G. M. Maelandsmo, *PLoS One* **2013**, *8*, e52980.
[4] G. Basurto-Islas, I. Grundke-Iqbal, Y. C. Tung, F. Liu, K. Iqbal, *Journal of Biological Chemistry* **2013**, *288*, 17495-17507.
[5] Y. Morita, H. Araki, T. Sugimoto, K. Takeuchi, T. Yamane, T. Maeda, Y. Yamamoto, K. Nishi, M. Asano, K. Shirahama-Noda, M. Nishimura, T. Uzu, I. Hara-Nishimura, D. Koya, A. Kashiwagi, I. Ohkubo, *FEBS Lett* **2007**, *581*, 1417-1424.
[6] E. Dall, H. Brandstetter, *Acta Crystallogr Sect F Struct Biol Cryst Commun* **2012**, *68*, 24-31.
[7] R. Breitling, S. Klingner, N. Callewaert, R. Pietrucha, A. Geyer, G. Ehrlich, R. Hartung, A. Muller, R. Contreras, S. M. Beverley, K. Alexandrov, *Protein Expr Purif* **2002**, *25*, 209-218.
[8] E. Dall, H. Brandstetter, *Proc Natl Acad Sci U S A* **2013**, *110*, 10940-10945.
[9] U. B. Ericsson, B. M. Hallberg, G. T. Detitta, N. Dekker, P. Nordlund, *Anal Biochem* **2006**, *357*, 289-298.
[10] F. Niesen, *Vol. 3.0*, 3.0 ed. (Ed.: F. Niesen), Structural Genomics Consortium, Oxford, **2010**.
[11] Z. Jia, S. Hasnain, T. Hirama, X. Lee, J. S. Mort, R. To, C. P. Huber, *J Biol Chem* **1995**, *270*, 5527-5533.
[12] T. G. Battye, L. Kontogiannis, O. Johnson, H. R. Powell, A. G. Leslie, *Acta Crystallogr D Biol Crystallogr* **2011**, *67*, 271-281.
[13] M. D. Winn, C. C. Ballard, K. D. Cowtan, E. J. Dodson, P. Emsley, P. R. Evans, R. M. Keegan, E. B. Krissinel, A. G. Leslie, A. McCoy, S. J. McNicholas, G. N. Murshudov, N. S. Pannu, E. A. Potterton, H. R. Powell, R. J. Read, A. Vagin, K. S. Wilson, *Acta Crystallogr D Biol Crystallogr* **2011**, *67*, 235-242.
[14] J. D. Thompson, D. G. Higgins, T. J. Gibson, *Nucleic Acids Res* **1994**, *22*, 4673-4680.
[15] N. Stein, *Journal of Applied Crystallography* **2008**, *41*, 641-643.
[16] A. J. McCoy, R. W. Grosse-Kunstleve, P. D. Adams, M. D. Winn, L. C. Storoni, R. J. Read, *J Appl Crystallogr* **2007**, *40*, 658-674.
[17] P. Emsley, K. Cowtan, *Acta Crystallogr D Biol Crystallogr* **2004**, *60*, 2126-2132.
[18] P. V. Afonine, R. W. Grosse-Kunstleve, N. Echols, J. J. Headd, N. W. Moriarty, M. Mustyakimov, T. C. Terwilliger, A. Urzhumtsev, P. H. Zwart, P. D. Adams, *Acta Crystallogr D Biol Crystallogr* **2012**, *68*, 352-367.
[19] P. D. Adams, R. W. Grosse-Kunstleve, L. W. Hung, T. R. Ioerger, A. J. McCoy, N. W. Moriarty, R. J. Read, J. C. Sacchettini, N. K. Sauter, T. C. Terwilliger, *Acta Crystallogr D Biol Crystallogr* **2002**, *58*, 1948-1954.
[20] G. N. Murshudov, A. A. Vagin, E. J. Dodson, *Acta Crystallogr D Biol Crystallogr* **1997**, *53*, 240-255.
[21] R. A. Laskowski, D. S. Moss, J. M. Thornton, *J Mol Biol* **1993**, *231*, 1049-1067.
[22] I. W. Davis, A. Leaver-Fay, V. B. Chen, J. N. Block, G. J. Kapral, X. Wang, L. W. Murray, W. B. Arendall, 3rd, J. Snoeyink, J. S. Richardson, D. C. Richardson, *Nucleic Acids Res* **2007**, *35*, W375-383.
[23] S. W. Ginzinger, M. Gruber, H. Brandstetter, M. J. Sippl, *Structure* **2011**, *19*, 1739-1743.
[24] W. L. DeLano, *DeLano Scientific, San Carlos, Ca, USA* **2002**.

[25] E. Krissinel, K. Henrick, *J Mol Biol* **2007**, *372*, 774-797.
[26] M. J. Sippl, *Bioinformatics* **2008**, *24*, 872-873.
